# The effect of osteopathic medicine on pain in musicians with nonspecific chronic neck pain: a randomized controlled trial

**DOI:** 10.1177/1759720X20979853

**Published:** 2020-12-10

**Authors:** Gabriele Rotter, Isabel Fernholz, Sylvia Binting, Theresa Keller, Stephanie Roll, Benjamin Kass, Thomas Reinhold, Stefan N. Willich, Alexander Schmidt, Benno Brinkhaus

**Affiliations:** Institute for Social Medicine, Epidemiology and Health Economics, Charité - Universitätsmedizin Berlin, corporate member of Freie Universität Berlin, Humboldt-Universität zu Berlin, and Berlin Institute of Health, Luisenstrasse 57, Berlin, 10117, Germany; Kurt-Singer-Institute for Music Physiology and Musicians Health, Hanns Eisler School of Music Berlin, Germany; Berlin Center for Musicians’ Medicine, Charité - Universitätsmedizin Berlin, corporate member of Freie Universität Berlin, Humboldt-Universität zu Berlin, and Berlin Institute of Health, Germany; Kurt-Singer-Institute for Music Physiology and Musicians Health, Hanns Eisler School of Music Berlin, Germany; Berlin Center for Musicians’ Medicine, Charité - Universitätsmedizin Berlin, corporate member of Freie Universität Berlin, Humboldt-Universität zu Berlin, and Berlin Institute of Health, Germany; Department of Psychiatry and Psychotherapy, Charité - Universitätsmedizin Berlin, corporate member of Freie Universität Berlin, Humboldt-Universität zu Berlin, and Berlin Institute of Health, Germany; Institute for Social Medicine, Epidemiology and Health Economics, Charité - Universitätsmedizin Berlin, corporate member of Freie Universität Berlin, Humboldt-Universität zu Berlin, and Berlin Institute of Health, Germany; Institute for Social Medicine, Epidemiology and Health Economics, Charité - Universitätsmedizin Berlin, corporate member of Freie Universität Berlin, Humboldt-Universität zu Berlin, and Berlin Institute of Health, Germany; Institute for Social Medicine, Epidemiology and Health Economics, Charité - Universitätsmedizin Berlin, corporate member of Freie Universität Berlin, Humboldt-Universität zu Berlin, and Berlin Institute of Health, Germany; Institute for Social Medicine, Epidemiology and Health Economics, Charité - Universitätsmedizin Berlin, corporate member of Freie Universität Berlin, Humboldt-Universität zu Berlin, and Berlin Institute of Health, Germany; Institute for Social Medicine, Epidemiology and Health Economics, Charité - Universitätsmedizin Berlin, corporate member of Freie Universität Berlin, Humboldt-Universität zu Berlin, and Berlin Institute of Health, Germany; Institute for Social Medicine, Epidemiology and Health Economics, Charité - Universitätsmedizin Berlin, corporate member of Freie Universität Berlin, Humboldt-Universität zu Berlin, and Berlin Institute of Health, Germany; Kurt-Singer-Institute for Music Physiology and Musicians Health, Hanns Eisler School of Music Berlin, Germany; Berlin Center for Musicians’ Medicine, Charité - Universitätsmedizin Berlin, corporate member of Freie Universität Berlin, Humboldt-Universität zu Berlin, and Berlin Institute of Health, Germany; Department of Audiology and Phoniatrics, Charité - Universitätsmedizin Berlin, corporate member of Freie Universität Berlin, Humboldt-Universität zu Berlin, and Berlin Institute of Health, Germany; Institute for Social Medicine, Epidemiology and Health Economics, Charité - Universitätsmedizin Berlin, corporate member of Freie Universität Berlin, Humboldt-Universität zu Berlin, and Berlin Institute of Health, Germany

**Keywords:** complementary medicine, musicians, neck pain, osteopathic medicine, randomized controlled trial

## Abstract

**Background:**

Nonspecific chronic neck pain (cNP) is common in adult violinists and violists and is often treated with osteopathic medicine (OM), although the effectiveness of this treatment has not been determined to date. This study aimed to evaluate the effectiveness and safety of OM in adult violinists and violists with cNP.

**Methods:**

In a two-armed randomized controlled single-center open trial, adult violinists and violists, including music students, with cNP (⩾12 weeks) were randomized to either five individualized OM sessions (OM group) or to no intervention (control group, CG) in the outpatient clinic for integrative medicine, Charité - Universitätsmedizin Berlin, Germany. All patients received a musicians’ medicine consultation and paracetamol on demand. The primary outcome parameter was the neck pain intensity on a visual analog scale (VAS, 0–100 mm, 0 = no pain, 100 = worst imaginable pain) after 12 weeks. Secondary outcomes included neck pain disability (Neck Disability Index, NDI, 0–100%) after 12 weeks. The last follow-up visit was after 52 weeks. Statistical analysis included analysis of covariance adjusted for respective baseline value.

**Results:**

Altogether, 62 outpatients were included [OM group (*n* = 28), CG (*n* = 34); 81% female; mean age, 41.6 ± 11.1 years; mean baseline neck pain, 55.9 ± 11.6 mm]. After 12 weeks, OM was associated with an improvement in the OM group *versus* the CG in neck pain on the VAS [14.6 mm (95% confidence interval 8.0; 21.2) *versus* 40.8 mm (34.7; 46.9), *p* < 0.001, Cohen’s *d* = 1.4], and neck pain disability as determined by the NDI [8.8% (6.7; 10.8) *versus* 17.2% (15.3; 19.1), *p* < 0.001]. Some improvements were maintained until 52 weeks of follow-up. No serious adverse events were observed.

**Conclusions:**

The results of this study suggest that OM might be effective in reducing pain intensity in adult violinists and violists with nonspecific cNP. Further studies should investigate the efficacy of OM in comparison with a sham procedure and with other effective therapy methods in high-quality multicenter trials.

**Trial registration:**

WHO Trial Registration https://apps.who.int/trialsearch/NoAccess.aspx?aspxerrorpath=/trialsearch/Trial2.aspx by German Clinical Trials Register DRKS00009258, Universal Trial Number (UTN): U1111-1173-5943.

## Introduction

Neck pain is a global burden and is reported to be a leading cause of ill health.^1^ For the purpose of the study, nonspecific chronic neck pain (cNP) was defined as pain in the anatomic region limited cranially by the superior nuchal line, caudally by the first thoracic vertebra, and laterally by the shoulder joint approaches of the trapezius muscle,^2^ not caused by pathologic findings, and with a symptom duration of at least 12 weeks. A multimodal approach, including manual treatments, advice, muscular stretching and exercise can be used to address cNP.^3–9^ Approximately 80% of professional musicians, including music students, experience health problems during their career that affect their performance, particularly neck pain and low back pain.^10–17^ For the purpose of the study, adult violinists and violists are professional musicians who perform in orchestras or as soloists and earn their living by making music or are music students playing the violin or viola. The high prevalence of neck pain^18^ in adult violists and violinists is attributed to the special playing demands, including frequent complex repetitive movements with long static and dynamic loads on the muscles in an asymmetric playing posture.^19^ Violists and violinists hold their instrument between the chin and shoulder, often using a shoulder rest and/or a chin rest to support this position. The left hand holds the instrument, the left fingers need to move freely to precisely pinch the note, performing fast and repetitive movements between a high position and a low position, while the right arm engages in repetitive bowing.^19,20^ Further risk factors include excess muscle tension, muscle fatigue, insufficient rest, long practice sessions, repertoire scheduling, poor posture, stress, poor injury management, performance anxiety, lack of fitness and insufficient warm-up.^14,21^ These risk factors can be addressed by prevention as practiced in musicians’ medicine.^22^ In adult musicians, only a few controlled intervention studies addressing musculoskeletal pain relief can be found.^16,23^ However, in musicians with cNP, trigger point therapy has been reported to be effective in pain reduction and functional improvement.^24^ In violists and violinists, manual treatments combined with musicians’ medicine have, to the best of the knowledge of the authors, been published only in case reports.^25,26^

Osteopathy and osteopathic medicine (both summarized in this paper under OM) are part of complementary and integrative medicine. OM is used by musicians for musculoskeletal symptoms.^23^ OM relies on manual contact for diagnosis and treatment and focuses on the structural and functional integrity of the body, including skeletal, arthrodial and myofascial structures and related vascular, lymphatic and neural elements^27^ in the so-called musculoskeletal, visceral and craniosacral systems. OM is commonly administered as a diagnosis-related and individualized treatment and additionally offers advice on self-training for postural improvement,^28^ as is performed in this study. A previous systematic review and meta-analysis in the general population indicated effectiveness for pain reduction with OM compared with heterogeneous comparison interventions, including physiotherapy, sham manipulation or no specific intervention, in patients with cNP.^29^

For patients receiving complementary and integrative medicine, pain reduction and lower costs have been reported from a hospital perspective in a noncontrolled retrospective analysis.^30^ Previous studies have indicated that OM may be cost effective for the management of neck pain in the general population; however, the published comparative effectiveness and health economics studies are of insufficient quality and quantity to draw further conclusions.^31–34^ To our knowledge, studies investigating the effectiveness, safety, costs, or cost effectiveness of OM in musicians with cNP are not available.^35^ The primary study aim was to evaluate the effect of five OM treatments in comparison with no OM treatment during 12 weeks on the subjectively perceived neck pain intensity in adult violinists and violists, including music students, with cNP. Further aims were to explore the impact of such therapy on the neck pain disability, stress intensity, quality of life, intake of analgesics, days of inability to work, days with restrictions in daily routine safety and cost effectiveness.

## Methods

### Study design

In a two-armed randomized controlled single-center open clinical trial, adult violinists and violists, including music students, with cNP were randomized to either five individualized OM sessions within 10 weeks (OM group) or to no OM intervention (control group, CG). All patients equally received a musicians’ medicine consultation addressing playing-related problems and paracetamol on demand.

### Setting

The study was performed at the outpatient clinic for integrative medicine of the Charité – Universitätsmedizin in Berlin, Germany, between September 2015 (first patient in) and May 2018 (last patient out after 52 weeks of follow-up).

This study was registered at the German Clinical Trials Register before enrollment of the first patient (DRKS00009258) and followed the standards of the Declaration of Helsinki^36^ and the ICH-GCP guidelines.^37^ It was approved by the Ethics Committee, Charité – Universitätsmedizin Berlin (approval number EA 1/198/15, with no amendments or any changes made to the study design). All patients gave oral and written informed consent before inclusion in the study.

### Patients

Patients were recruited from various professional orchestras in Berlin and nearby federal states (Brandenburg, Mecklenburg-Western Pomerania, Saxony, Saxony-Anhalt, and Thuringia) and Berlin music schools/universities. We used posters, flyers, newspapers, electronic listings and digital media for recruiting. Violinists and violists who were active professional orchestral musicians, soloists, or music students of both sexes aged 18–65 years with a clinical diagnosis of cNP for at least 12 weeks prior to study onset and an average pain intensity within the last 7 days of at least 40 mm on a 100-mm horizontal visual analog scale (VAS, 0 = no pain, 100 = worst imaginable pain, Supplement 1) were included in the study. A 40-mm cutoff point for study inclusion was selected by the study team based on the literature, including a randomized controlled trial (RCT) investigating OM in patients with cNP^38^ and earlier pain studies in our group.^39,40^ A 40-mm cutoff point allows defining a population with at least medium pain severity to provide some homogeneity in pain intensity and to make recruitment of the study population feasible. Within the last 4 weeks before the start of the study, patients had used no therapy or only drug therapy for cNP. The exclusion criteria were defined as follows: peripheral or central neurological symptoms; known vascular anomaly, such as aneurysm; known or suspected primary or secondary bone tumor; neck pain caused by recent trauma; rheumatic disease; prior surgery on the cervical column; suspected osteoporosis; OM treatment within the last 6 months before the beginning of the study; neck pain treated by complementary medicine or physical therapy within the last 3 months before inclusion; obesity (body mass index > 30 kg/m^2^); current intake of centrally acting analgesics; pregnancy; presence of other acute or chronic disease impairing participation in the study intervention; presence of other psychic or somatic condition impairing participation in the study intervention; alcohol or substance abuse; planned or actual use of therapy with possible impact on cNP, such as physiotherapy, acupuncture, massage, neuroreflex therapy, or the Feldenkrais method, during study participation; insufficient German language skills; current application for a benefit; and participation in another clinical trial during the 6 months before the study or parallel to the study.

Patients were randomized to one of the two treatment groups (1:1 ratio) by a computer-generated block randomization process in the study center with variable block length. The allocation was performed in the study center by a study nurse and was concealed.

## Study intervention

### Both groups

Before randomization, all patients received a 45-minute semi-standardized musicians’ medicine consultation addressing playing-related problems with one of two experts in musicians’ medicine (IF, AS), supported by a handout. The musicians’ medicine consultation and handout were established by a consensus of experts, which included a review of the literature.^16^ The musicians’ medicine consultation and handout addressed risk factors for cNP in violinists and violists, especially excess muscle tension, muscle fatigue, insufficient rest, long practice sessions, repertoire scheduling, poor posture, stress, poor injury management, performance anxiety, lack of fitness and insufficient warm-up.^14^ The consultation included behavioral advice regarding playing practice, lifestyle recommendations, instrument-specific ergonomics and occupational environment and advice for the work organization, which included the number of working hours and sufficient breaks. The details of the handout are provided in the supplemental material online (Supplement 2).

All patients were allowed to take 500 mg of paracetamol on demand up to four times daily during the first 12 weeks. Twelve weeks after randomization, patients in both groups were allowed to use any additional physical or psychological treatment.

### OM group

Within the first 12 weeks after randomization, patients in the OM group received five individualized diagnosis-related OM treatment sessions that were 45 min long each at an approximately 2-week interval. Each session started with a short interview and physical examination of the musculoskeletal, visceral and craniosacral systems according to medical and OM principles. Based on the interview and physical examination, the actual treatment strategy following OM principles was determined for each session. According to the individually necessary treatment techniques in the musculoskeletal, visceral and craniosacral systems, patients were treated in a sitting or lying position with or without active participation of the patient. We chose an individualized diagnosis-related OM treatment approach, as is commonly administered in OM. Advice for postural improvement during instrument playing was included in the OM treatment. Therefore, all musicians were examined during one of the five OM treatment sessions while playing the instrument. The study intervention was applied by one medical doctor (and osteopath) with special expertise in musicians’ medicine (GR).

### Control group

Patients in the CG received no OM treatment within the first 12 weeks. After 12 weeks, patients of the CG could receive five OM treatments free of charge, if desired.

## Outcome parameters and data collection

Parameters were measured at baseline and after 6, 12, 26, and 52 weeks using standardized patients’ questionnaires. The primary outcome was neck pain; patients rated their average perceived neck pain within the last 7 days on a horizontal VAS (0–100 mm, 0 = no pain, 100 = worst imaginable pain, Supplement 1) after 12 weeks.^41^ We used the VAS for pain measurement because it is validated, widely used, easy to use and takes less than 1 min to complete.^41,42^ Recently, the minimal clinically important difference (MCID) on the VAS for neck pain was reported to be within a range between 4.6 mm and 21.4 mm.^43^ During the planning of the study, we considered publications with an MCID of 13.7 mm on a 100-mm VAS for pain measurement,^42^ of 8 mm on a 100-mm VAS for neck pain,^44^ and of 1.5 points (range, 1–10) on a numeric rating scale for neck pain.^45^ Based on the literature and our expectations for our study population, we decided to select an MCID of 15 mm on a 100-mm VAS to measure neck pain. The criterion for a substantial clinical benefit (SCB) was reported to be 26.5 mm.^44^

VAS neck pain levels after 6, 26, and 52 weeks were considered secondary outcomes. A further secondary outcome after 6, 12, 26 and 52 weeks was neck pain disability assessed by the Neck Disability Index (NDI, 0–100%) in a validated German version.^46,47^ The NDI^46^ measures neck pain disability in everyday life. The NDI is widely used and well validated.^48–52^ The NDI is easy for the patient to fill out and easy for the investigator to evaluate. For the NDI, the MCID is given between 3.0 and 9.5 points (0–50 point scale)^44,53,54^ or 9.8% (0–100% scale,^44^ used in the present study) with an SCB of 29%.^44^ Further secondary outcomes were stress intensity as determined by a horizontal VAS for stress. We used the VAS for stress measurement because it is validated,^55^ easy to use and takes less than 1 min to complete. Patients rated their average perceived stress within the last 7 days on a horizontal VAS (0–100 mm, 0 = no stress, 100 = worst imaginable stress),^55^ with no MCID for VAS stress determined, and health-related quality of life measured by the 12-item Short Form Health Survey (SF-12, MCID: 5 points).^56–59^ We used the SF-12^58,59^ because it is a short form of the well-accepted SF-36^56,57^ and is commonly used.

Furthermore, we assessed the intake of analgesics in a diary, which we applied despite reported tendencies toward inaccuracy regarding the time and reliability of entries,^60^ the days of inability to work due to cNP within the last 12 weeks (baseline) and within the last 6 weeks (all other measurement points), and the days with restriction in daily routine due to cNP within the last 12 weeks for baseline and within the last 6 weeks (all other measurement time points) by the (not validated) question: “On how many days in the last 12 (respective 6) weeks have you been restricted in your daily routine due to cervical spine pain?” Safety (adverse events and serious adverse events) was assessed by the study physician during the interviews in the OM group; additionally, patients were encouraged to contact the study center in the case of any adverse events. Patients also rated the changes in their complaints due to musculoskeletal pain within the last 6 weeks, and patients receiving OM rated the effectiveness of the OM treatment with reference to the last 6 weeks (“highly effective,” “effective,” “slightly effective,” “not effective”). Sociodemographic data, including age, sex, and education, were also assessed at baseline.

We decided to apply patient-relevant outcomes by using patient-reported outcome measures and not to add objective parameters. Therefore, blinding of outcome assessors (patients) was not feasible.

## Statistical analysis

Sample size: For the primary outcome (VAS score of neck pain after 12 weeks), the sample size was calculated based on a consensus considering a previous German RCT in OM for cNP,^38^ a Berlin RCT investigating Tui Na in cNP,^61^ and an older pre–post pilot study investigating OM in patients with cNP and subchronic neck pain,^62^ including literature about the MCID for the VAS for pain, ranging from at 8 mm in patients with cNP^44^ to 13.7 mm for pain,^42^ and including the MCID of 1.5 points (0–10) on a numeric rating scale.^45^ As a result, a mean difference between the OM group and the CG of 15 mm was considered,^38^ and the common standard deviation was assumed to be 25 mm. Thus, with 45 patients per group (90 in total), a two-sided *t*-test with a significance level of 5% would have a power of 80%. To compensate for potential dropouts, 100 patients were intended to be randomized (50 patients per group).

The primary analysis of the primary outcome was performed using an analysis of covariance (ANCOVA) with a fixed-factor treatment group adjusted for the baseline value of the VAS score for neck pain. The assumptions for normal distribution were both tested with reviewing histograms and Q–Qplots. The assumptions of equal regression slopes, outliers and linearity were checked with scatter plots, the assumption of variance homogeneity was tested with the Levene’s test. The significance level was established as <5% (*p* < 0.05). Post hoc Cohen’s *d* was calculated for the VAS score of neck pain after 12 weeks. Cohen’s *d* and all following analyses were considered explorative. The secondary outcomes for the VAS score of neck pain (after 6 weeks), neck pain disability as determined by the NDI, VAS score for stress, SF-12, quality-adjusted life years (QALYs), and total costs over the first 12 weeks were analyzed similar to the analysis of the primary outcome, that is, by ANCOVA adjusted for the respective baseline values. The results are reported as adjusted group means with 95% confidence intervals (CIs) and the *p*-value for the treatment group comparison. The *p*-values are only reported for the first 12 weeks as participants in the CG also received OM after week 12. All tests and CIs were two sided. All data were analyzed based on the intention-to-treat-principle using the full analysis set (FAS) with all available data without imputing missing data. All analyses were performed according to the original assigned groups. Adverse events are presented descriptively by frequency for each treatment group. In addition, a number of sensitivity analyses were performed. A per-protocol (PP) analysis was performed for the primary outcome, excluding patients if at least one of the following criteria was met: no complete data available for the primary endpoint, namely, the VAS score for neck pain at 12 weeks; not treated according to the allocated group; fewer than five interventions in the first 12 weeks (OM group only); and OM treatment (elsewhere) during the first 12 weeks (CG only).

Further, a sensitivity analysis of the VAS score for neck pain, neck pain disability as determined by the NDI, VAS score for stress and SF-12 was performed by ANCOVA adjusted for respective baseline values and for sex, education and the VAS score for stress. A responder criterion in a range of 30–50% pain reduction has been used in trials^40,63^ and is recommended for research.^64^ Furthermore, a pain reduction of 50% was reported to be meaningful.^65^ Based on this literature, a responder was defined by at least 50% pain reduction as determined the VAS score for neck pain, and a post hoc responder analysis was performed. Statistical analyses, including health economics analyses, were performed using the software package SAS 9.4.^66^

## Health economics analysis

In addition, a cost-effectiveness analysis was carried out for the period 12 weeks after baseline. Therefore, the achieved QALYs were linked to cNP-related total costs from a societal perspective (including direct and indirect costs). Data on utilization of medical resources, sick leave days and working hour reductions related to cNP were systematically collected using patient questionnaires and valued by using standardized German national unit cost assumptions. Costs arising due to the OM intervention were considered to be 85.80 Euro per session, according to a notification from the study center financial department. An algorithm developed by Brazier and Roberts^67^ was applied to convert the data of the SF-12 into the SF-6D health state utility values. QALYs were measured based on these utility values by calculating the area under the curve, assuming linear changes between the longitudinal utility values. In the case of a significant QALY gain and significant additional costs in the OM group, it was planned to calculate the incremental cost-effectiveness ratio (ICER), reflecting the add-on costs for realizing one QALY gained.

## Results

### Patients and treatment

**Figure 1.**
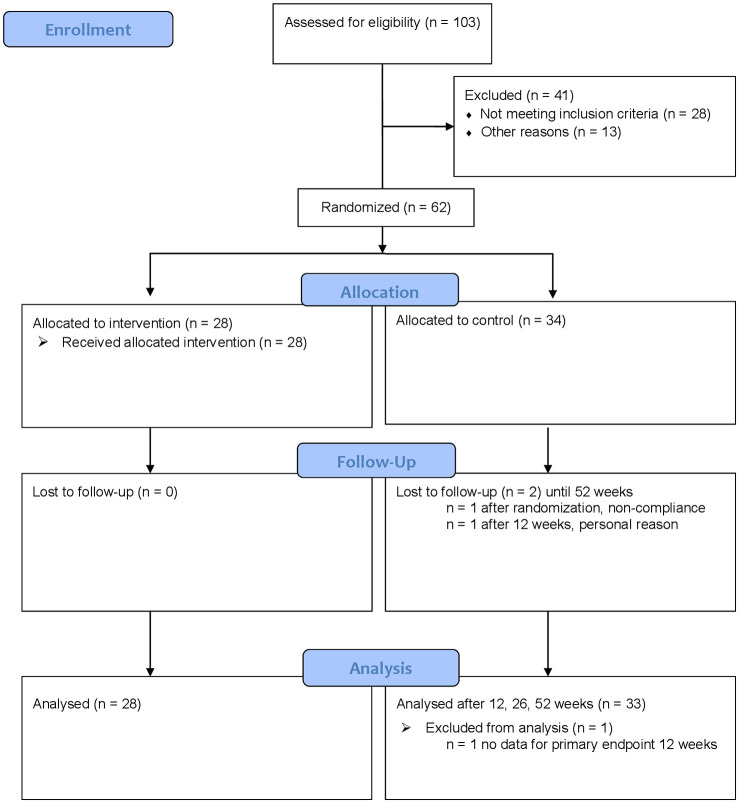
Recruitment, treatment and follow-up of patients with chronic neck pain.

From 103 eligible patients, 62 were enrolled between September 2015 and May 2017 and were randomized into the two treatment groups (OM group, *n* = 28; CG, *n* = 34). Despite strong efforts, it was not possible to include more patients in the study, and the targeted sample size of *n* = 100 was not reached. After randomization, one patient in the CG dropped out due to noncompliance; after 12 weeks, another patient in the CG dropped out due to personal reasons. All other patients remained completed 52 weeks of follow-up and returned the questionnaires (Figure 1). After 12 weeks, 28 (82.3%) patients in the CG received at least one session of OM (anytime within the 52 weeks of follow-up and without restrictions regarding the treatment interval).

At baseline, the mean age was 41.6 ± 11.1 years (mean ± standard deviation). Fifty patients (80.7%) were female. The mean duration of cNP symptoms was 14.0 ± 10.9 years in the OM group and 13.8 ± 9.5 years in the CG (Table 1). At baseline, there were relevant differences between the OM group and the CG regarding education level (German university entrance qualification: OM group, 67.9%; CG, 91.2%), VAS scores for stress (OM group, 44.4 mm ± 22.9; CG, 57.8 mm ± 18.3) and total costs over the 12 weeks prior to baseline (OM group, 750.05 ± 2,013.18 EUR; CG, 370.03 ± 673.19 EUR). The mean VAS score for neck pain was 56.9 ± 11.6 mm in the OM group and 55.0 ± 11.7 mm in the CG. Thirty (48.4%) patients had received osteopathic treatment (mostly by nonphysician osteopaths) in their life before, including 12 (42.9%) patients in the OM group and nine (26.5%) patients in the CG, because of cNP. Apart from cNP, further self-perceived health problems were reported by 24 (85.7%) patients in the OM group and 32 (94.1%) patients in the CG; the most frequently reported issue was shoulder pain [*n* = 20 (83.3%) patients in the OM group, *n* = 26 (81.3%) patients in the CG]. The physical examination at baseline revealed pathologic findings in the musculoskeletal system in 11 (39.3%) patients in the OM group and 9 (26.5%) patients in the CG.

**Table 1.** Baseline characteristics of patients.

	Osteopathic medicine group *n* = 28 Mean ± SD/*n* (%)	Control group *n* = 34 Mean ± SD/*n* (%)	Total *n* = 62 Mean ± SD/*n* (%)
Age (years)	42.8 ± 11.5	40.6 ± 10.8	41.6 ± 11.1
Range	21–63	21–63	21–63
Sex (female)	23 (82.1)	27 (79.4)	50 (80.7)
BMI (kg/m^2^)	23.2 ± 3.3	22.0 ± 2.5	22.6 ± 2.9
Physically active	20 (71.4)	25 (73.5)	45 (72.6)
German university entrance qualification (Abitur)^***^	19 (67.9)	31 (91.2)	50 (80.7)
Employed (in students: in addition to study) (yes)	26 (92.9)	33 (97.1)	59 (95.2)
If employed (*n* = 59), incapacity for work last 12 weeks (days)	1.0 ± 3.1	0.7 ± 1.6	0.8 ± 2.3
Min–Max	0–14	0–7	0–14
Background^a^			
Professional musician	26 (92.9)	32 (94.1)	58 (93.6)
Student	2 (7.1)	3 (8.8)	5 (8.1)
Main instrument			
Violin	19 (67.9)	26 (76.5)	45 (72.6)
Viola	8 (28.6)	8 (23.5)	16 (25.8)
Both	1 (3.6)	0	1 (1.6)
Orchestra part			
Solo	4 (15.4)	4 (12.5)	8 (13.8)
Tutti	23 (88.5)	30 (93.8)	53 (91.4)
Time of playing last 6 weeks (average hours/day)	3.3 ± 1.9	3.5 ± 1.6	3.4 ± 1.7
Time of practice last 6 weeks (average hours/day) (*n* = 61^b^)	1.7 ± 1.2	2.0 ± 2.0	1.9 ± 1.6
Duration of cNP (years)	14.0 ± 10.9	13.8 ± 9.5	13.9 ± 10.1
Min–Max	1.0–40.0	0.3–40.0	0.3–40.0
Pathologic findings in musculoskeletal system	11 (39.3)	9 (26.5)	20 (32.3)
Osteopathic treatment earlier	13 (46.4)	17 (50.0)	30 (48.4)
Osteopathic treatment earlier because of cNP	12 (42.9)	9 (26.5)	21 (33.9)
VAS neck pain (0–100 mm)^*^	56.9 ± 11.6	55.0 ± 11.7	55.9 ± 11.6
Neck pain disability by NDI (0–100%)^*^	20.6 ± 7.9	20.6 ± 7.6	20.6 ± 7.7
VAS stress (0–100 mm)^*^, ^***^	44.4 ± 22.9	57.8 ± 18.3	51.7 ± 21.4
SF-12 Physical Component Scale (0–100)^**^ (*n* = 60^b^)	47.0 ± 8.0	46.7 ± 7.6	46.8 ± 7.7
SF-12 Mental Component Scale (0–100)^**^ (*n* = 60^b^)	46.3 ± 11.1	44.7 ± 9.2	45.4 ± 10.0
Days with restriction in activities of daily living last 12 weeks	21.4 ± 25.3	22.7 ± 27.2	22.1 ± 26.1
Min–Max	0–90	0–92	0–92
Restriction in making music due to cNP last 6 weeks (*n* = 61^b^)	11 (39.3)	19 (57.6)	30 (49.2)
Days	20.0 ± 13.7	29.8 ± 14.4	26.4 ± 14.7
Min–Max (*n* = 29)	4–42	7–42	4–42
Satisfaction with working atmosphere			
Very satisfied	5 (17.9)	5 (15.6)	10 (16.7)
Satisfied	18 (64.3)	14 (43.8)	32 (53.3)
Neutral	4 (14.3)	12 (37.5)	16 (26.7)
Dissatisfied	1 (3.6)	1 (3.1)	2 (3.3)
Very dissatisfied	0	0	0
Study physician expectation of OM intervention			
Cure	0	0	0
Significant recovery	16 (57.1)	14 (41.2)	30 (48.4)
Slight recovery	12 (42.9)	20 (58.8)	32 (51.6)
No recovery	0	0	0
Patients expectation of OM intervention			
Cure	0	6 (17.7)	6 (9.7)
Significant recovery	26 (92.9)	25 (73.5)	51 (82.3)
Slight recovery	2 (7.1)	3 (8.8)	5 (8.1)
No recovery	0	0	0
Direct costs of cNP last 12 weeks (EUR)	67.69 ± 221.49	72.22 ± 177.24	70.17 ± 196.76
Indirect costs of cNP last 12 weeks (EUR)	682.36 ± 1,967.04	297.80 ± 614.38	471.48 ± 1,397.87
Total costs of cNP last 12 weeks (EUR)^***^	750.05 ± 2,013.18	370.03 ± 673.19	541.65 ± 1,440.63

a more than one answer possible: student and professional musician.

b baseline data only for *n *= respective value available.

* lower values indicate better status.

** higher values indicate better status.

*** relevant differences between groups.

BMI, Body Mass Index; cNP, chronic neck pain; Max, maximum; Min, minimum; *n*, number; NDI, Neck Disability Index; OM, osteopathic medicine; SD, standard deviation; SF-12, 12-item Short Form Health Survey; VAS, visual analog scale.

### Outcomes

After 12 weeks, the primary outcome, the VAS score for neck pain, was significantly and relevantly lower in the OM group [OM group-adjusted mean, 14.6 mm, 95% CI (8.0; 21.2); CG, 40.8 mm, (34.7; 46.9)] with an adjusted group difference of −26.2 mm [(−35.2; −17.2), *p* < 0.001] (Table 2). The effect size (Cohen’s *d*) for the VAS score for neck pain after 12 weeks was *d* = 1.4 (*d* = 1.5, if adjusted for the baseline VAS score for neck pain). The sensitivity analyses for baseline differences and PP analyses for the VAS score for neck pain, neck pain disability as determined by the NDI, VAS score for stress, and SF-12 were also similar to the above-reported FAS analyses. The responder analysis for 50% pain reduction after 12 weeks revealed a point estimate (odds ratio) of 13.8 [95% Wald CI (3.8−50.3), *p* < 0.001].

**Table 2.** Primary and main secondary outcomes until 12 weeks (intergroup comparison), and until 52 weeks, patients of the control group started to receive OM treatment after week 12.

	*n*	Osteopathic medicine group-adjusted mean, (95% CI)^a^	Control group-adjusted mean, (95% CI)^a^	Differences (osteopathic medicine group − control group) adjusted mean, (95% CI)^a^	*p*
VAS neck pain (0−100 mm)^*^ (average neck pain during the previous 7 days), MCID 15 mm, SBC 26.5 mm					
6 weeks	57	21.9 (14.7; 29.1)	42.8 (36.2; 49.4)	−20.9 (−30.7; −11.1)	<0.001
12 weeks (primary outcome)	61	14.6 (8.0; 21.2)	40.8 (34.7; 46.9)	−26.2 (−35.2; −17.2)	<0.001
26 weeks^***^	58	20.5 (12.0; 29.0)	35.8 (28.2; 43.5)	^***^	^***^
52 weeks^***^	56	19.7 (12.2; 27.7)	30.6 (23.8; 37.5)	^***^	^***^
Neck pain disability by NDI (0−100%)^*^, MCID 9.8%					
6 weeks	61	14.1 (11.8; 16.4)	18.6 (16.4; 20.7)	−4.5 (−7.7; −1.4)	0.006
12 weeks	61	8.8 (6.7; 10.8)	17.2 (15.3; 19.1)	−8.4 (−11.2; −5.6)	<0.001
26 weeks^***^	58	10.8 (8.0; 13.7)	15.1 (12.5; 17.6)	^***^	^***^
52 weeks^***^	57	10.3 (7.6; 13.0)	14.0 (11.5; 16.6)	^***^	^***^
VAS stress (0−100 mm)^*^					
6 weeks	61	40.7 (32.3; 49.0)	51.5 (43.8; 59.2)	−10.8 (−22.4; 0.8)	0.067
12 weeks	61	30.4 (21.6; 39.2)	46.1 (38.0; 54.2)	−15.7 (−27.9; −3.4)	0.013
26 weeks^***^	58	39.8 (28.6; 50.9)	44.2 (34.2; 54.2)	^***^	^***^
52 weeks^***^	57	35.0 (25.2; 44.8)	41.8 (32.4; 51.0)	^***^	^***^
SF-12 physical component scale (0–100)^**^, MCID 5 points					
6 weeks	59	51.2 (49.1; 53.3)	48.2 (46.3; 50.2)	3.0 (0.1; 5.8)	0.044
12 weeks	59	53.1 (51.2; 54.9)	49.1 (47.3; 50.8)	4.0 (1.5; 6.6)	0.003
26 weeks^***^	55	52.9 (50.6; 55.2)	49.7 (47.7; 51.8)	^***^	^***^
52 weeks^***^	54	51.7 (49.2; 54.1)	49.6 (47.2; 52.0)	^***^	^***^
SF-12 mental component scale (0–100)^**^, MCID 5 points					
6 weeks	59	48.0 (44.6; 51.2)	46.7 (43.7; 49.7)	1.3 (–3.1; 5.7)	0.563
12 weeks	59	49.8 (46.3; 53.3)	47.5 (44.4; 50.7)	2.0 (–1.4; 5.5)	0.335
26 weeks^***^	55	47.9 (44.3; 51.5)	47.6 (44.4; 50.8)	^***^	^***^
52 weeks^***^	54	49.3 (45.3; 53.3)	44.3 (40.5; 48.2)	^***^	^***^

a Results adjusted for respective baseline value.

* lower values indicate better status.

** higher values indicate better status.

*** no comparison and no *p*-values, because after 12 weeks *n* = 28 (82.4%) participants of the control group started to receive OM treatment.

CI, confidence interval; MCID, minimal clinically important difference; *n*, number for respective available data from *n* = 61 patients; NDI, Neck Disability Index; SBC, substantial clinical benefit; SF-12, 12-item Short Form Health Survey; VAS, visual analog scale.

**Figure 2.**
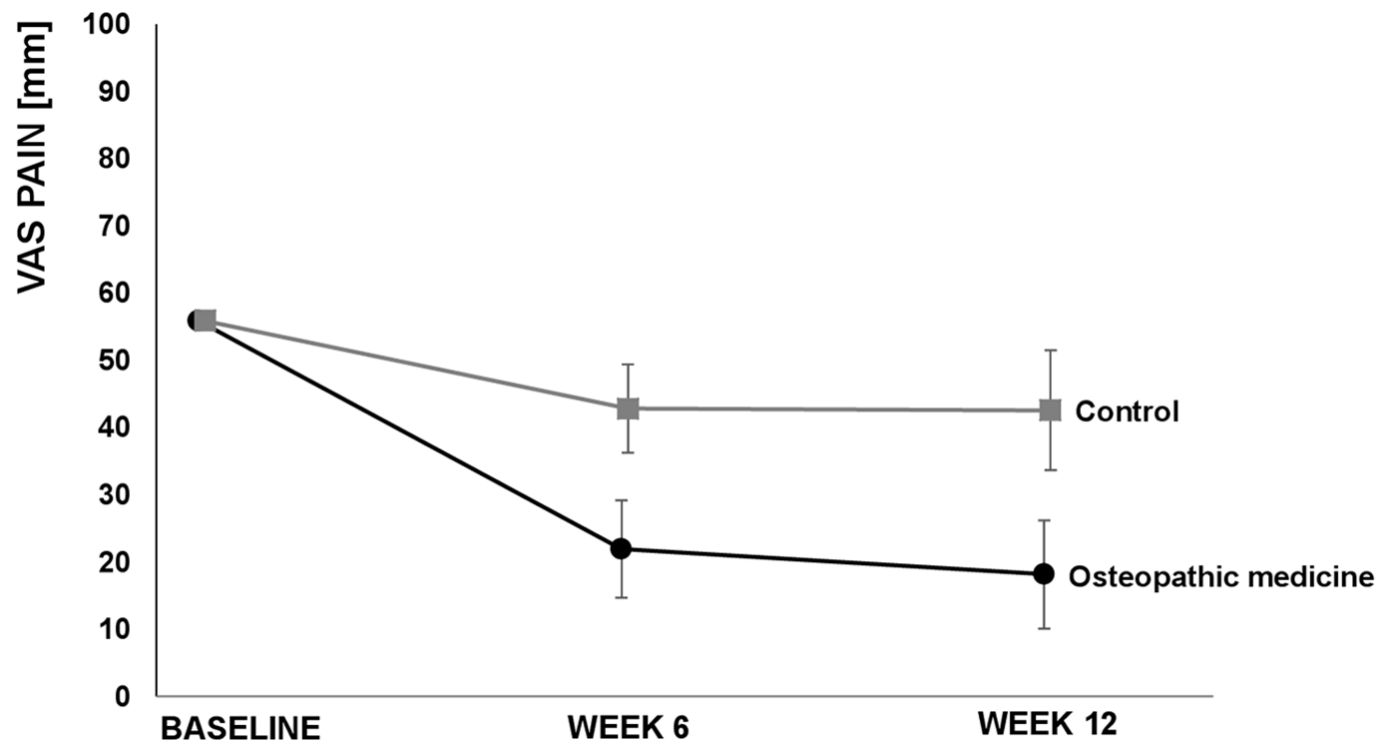
Primary outcome visual analog scale (VAS) pain over 12 weeks. Values are adjusted means and 95% confidence intervals.

**Figure 3.**
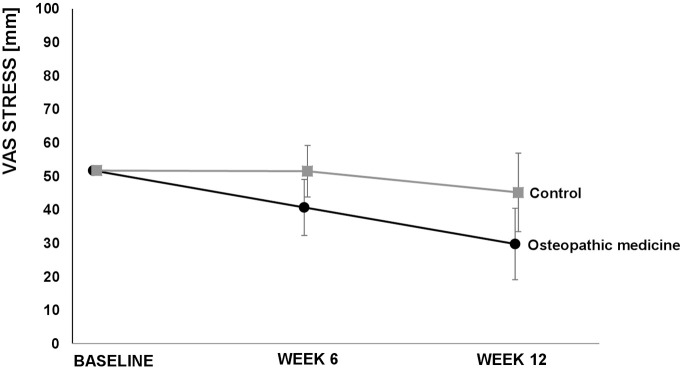
Visual analog scale (VAS) stress over 12 weeks. Values are adjusted means and 95% confidence intervals.

**Figure 4.**
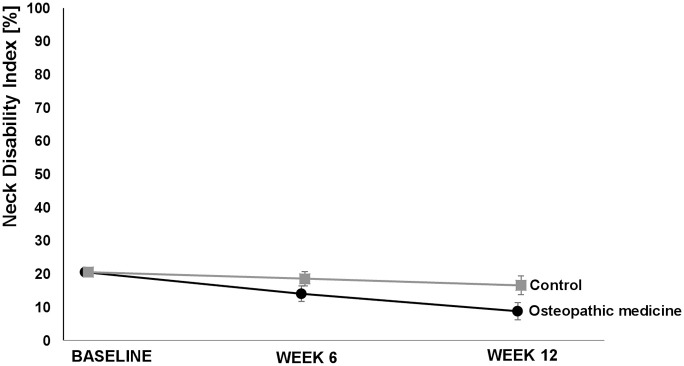
Neck disability index over 12 weeks. Values are adjusted means and 95% confidence intervals.

**Figure 5.**
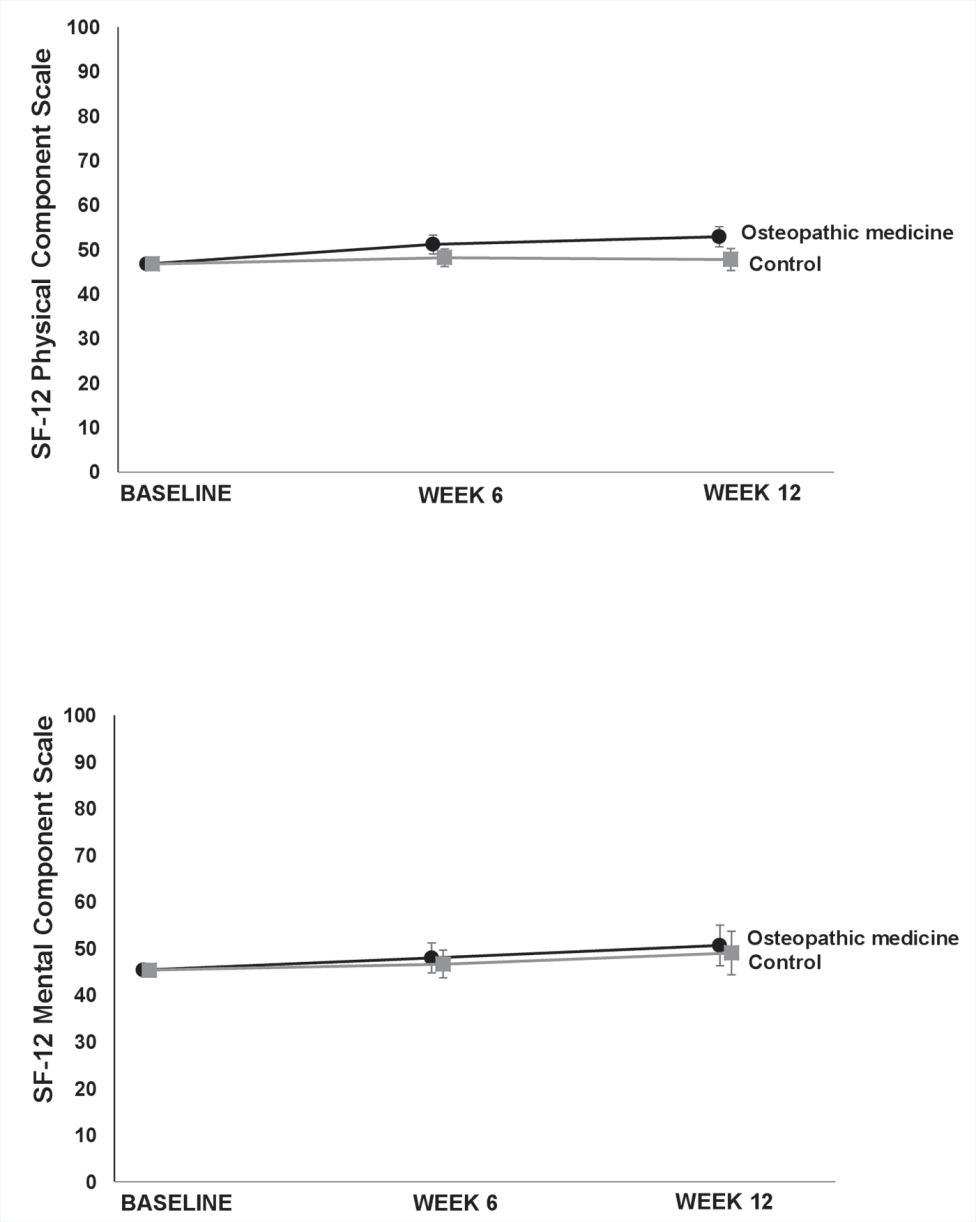
12-item Short Form Health Survey (SF-12) Physical Component Scale and Mental Component Scale over 12 weeks. Values are adjusted means and 95% confidence intervals.

The VAS score for neck pain after 6 weeks (secondary outcome) showed a clinically relevant difference favoring the OM group [−20.9, (−30.7; −11.1), *p* < 0.001]. Furthermore, we observed (not clinically relevant) differences in the baseline value-adjusted mean neck pain disability as determined by the NDI in favor of the OM group after 6 weeks [−4.5%, (−7.7; −1.4), *p* = 0.006] and 12 weeks [−8.4%, (−11.2; −5.6), *p* < 0.001]. The baseline value-adjusted mean VAS score for stress was lower in the OM group after 12 weeks [−15.7 mm, (−27.9; −3.4), *p* = 0.013] but not after 6 weeks [−10.8 (−22.4; 0.8), *p* = 0.067]. Regarding the SF-12, although better results in favor of OM were found for the baseline-adjusted mean difference for the physical component scale after 6 [3.0, (0.1; 5.8), *p* = 0.044] and 12 weeks [4.0, (1.5; 6.6), *p* = 0.003], the differences were not clinically relevant. There were no relevant effects on the mental component scale of the SF-12 in the OM group in comparison to the CG after 6 [1.3 95% CI (−3.1; 5.6), *p* = 0.5632] or 12 weeks [2.0 95% CI (−1.4; 5.5), *p* = 0.3351] (Table 2, Figures 2−5). For some outcomes, not all assumptions for ANCOVA were met. However, repeating the analysis correcting for the respective violation, the results from ANCOVA were robust (data not shown) and did not alter the interpretations.

After 12 weeks, 28 patients (82.4%) in the CG also received OM within the 52 weeks of follow-up. Among them, 24 patients (70.6%) received all five OM treatment sessions, and 27 patients (79.4%) received at least four OM treatment sessions within 52 weeks after baseline. After 52 weeks, patients in the OM group reported a VAS score for neck pain of 19.7 mm (12.2; 27.7), while patients in the CG reported a VAS score for neck pain of 30.6 mm (23.8; 37.5) (Table 2).

Analgesic use within the first 12 weeks was low overall and comparable between the two groups. In the OM group, only five patients (17.9%) took 24 pills of paracetamol in total, and in the CG, four patients (11.8%) took 23 pills of paracetamol in total. Regarding analgesics other than paracetamol, one patient in the OM group used arnica pain ointment, and others used 400−600 mg of ibuprofen or did not specify the dose of ibuprofen. In the OM group, two patients (7.1%) took a total of 16 analgesic doses (arnica pain ointment, ibuprofen). In the CG, four patients (11.8%) took 43 analgesic doses (ibuprofen) altogether.

Furthermore, we found a decrease in the days with inability to work due to cNP in the OM group within the first 12 weeks, which could not be observed in the CG. Days of inability to work due to cNP were measured at baseline (for the last 12 weeks) in the OM group and CG, with a mean of 1.0 (−0.2; 2.3), and 0.7 (0.1; 1.2), respectively; after 6 weeks (for the last 6 weeks) in the OM group and CG, the mean was 0.1 (−0.1; 0.3) and 0.3 (−0.1; 0.7), respectively; and after 12 weeks (for the last 6 weeks), in the OM group and CG, the mean was 0.0 (0.4; 3.2) and 0.5 (−0.2; 1.2), respectively.

Days with restrictions in daily routine due to cNP improved in the OM group *versus* the CG [difference, −6.1 (−10.2; −2.1), *p* = 0.004] within the first 12 weeks (Table 3). Patients further evaluated their complaints due to musculoskeletal pain. Patients in the OM group reported a better improvement than patients in the CG (Table, Supplement 3). Most patients in the OM group rated the intervention as very effective after 12 weeks.

**Table 3.** Days with restriction in daily routine due to chronic neck pain.

Days with restriction in daily routine due to chronic neck pain	*n*	Osteopathic medicine group, adjusted mean, (95% CI)^a^	Control group, adjusted mean, (95% CI)^a^	Differences (osteopathic medicine group − control group), adjusted mean, [95% CI]^a^	*p*
After 6 weeks (last 6 weeks)	61	4.0 (0.3; 7.6)	11.2 (7.8; 14.6)	−7.3 (−12.3; −2.2)	0.005
After 12 weeks (last 6 weeks)	60	1.8 (−1.2; 4.9)	8.0 (5.2; 10.7)	−6.1 (−10.2; −2.1)	0.004
After 26 weeks (last 6 weeks)^*^	57	1.4 (–1.8; 4.5)	7.0 (4.2; 9.8)	^*^	^*^
After 52 weeks (last 6 weeks)^*^	57	2.5 (−0.7; 5.7)	4.9 (1.9; 8.0)	^*^	^*^

a Results adjusted for respective baseline value.

* no comparison and no *p*-values, because after 12 weeks 28 (82.4%) participants of the control group started to receive OM treatment.

CI, confidence interval; *n*, number for respective available data from 61 patients.

No serious adverse events were observed. Two patients reported transient mild adverse events after OM (tiredness and dizziness).

During the first 12 weeks after baseline, the OM group experienced 0.1789 adjusted QALYs (0.1734; 0.1845) compared with patients in the CG, with 0.1734 QALYs (0.1682; 0.1785). The adjusted mean QALY difference was 0.0055 (−0.0020; 0.0132) (*p* = 0.147), in favor of the OM group. For the same time period, a mean adjusted cost of 497.54 EUR (2.56; 992.52) and 704.54 EUR (242.94; 1166.14) occurred in the OM group in the CG, resulting in an adjusted mean difference of −207.00 EUR [(−887.43; 473.43), *p* = 0.545], in favor of the OM group. The additional direct costs due to the OM intervention seemed to be mainly compensated for by lower indirect costs due to work absenteeism in the OM group. An ICER was not calculated since the differences in QALYs and total costs were not statistically significant between the groups.

## Discussion

Five OM sessions were associated with a clinically relevant and statistically significant reduction in the mean neck pain intensity in comparison to patients receiving no OM treatment. All further outcomes were exploratory and must be investigated in further confirmatory studies. Patients in the OM group tended to show improvements in neck pain disability and the SF-12 physical component compared with patients in the no-intervention CG, although the improvement was not clinically relevant. Furthermore, a tendency toward a reduction in the VAS score for stress was found in the OM group compared with the CG. Positive effects in the OM group were maintained, to some degree, for the mean neck pain intensity, neck pain disability and the SF-12 physical component scale within the 52 weeks of follow-up. The OM treatment was safe but not superior in terms of cost effectiveness.

We considered the adjusted group difference for the VAS score for neck pain, with –26.2 mm (with a large effect size, Cohen’s *d*) indicating a good clinical result, even if the criterion for an SCB at −26.5 mm^44^ was missed. The failure to meet the SCB criterion might be due to various factors: First, patients in the CG improved as well, which could be due to study effects and to the semi-standardized musicians’ medicine consultation, which was provided equally for both groups. The musicians’ medicine consultation included a variety of playing and performance-related information. In particular, posture, optimization of shoulder rest/chin holder, and practice scheduling were determined to be important factors of cNP among violinists and violists and can be addressed by musicians’ medicine.^14,19,22,68–72^ Overall, the improvement in pain in the CG during the first 12 weeks also indicates (in addition to study effects) that a musicians’ medicine consultation might be helpful.

We used a computer-generated block randomization method as it helps to prevent accidental bias and to achieve balance between groups.^73^ However, within trials with a smaller study population, there can be baseline differences despite randomization by chance. In order to adjust for the risk of baseline differences between the groups regarding educational level and the VAS score for stress, we performed sensitivity analyses with adjustments for these factors and additionally for sex regarding the primary outcome of the VAS score for neck pain. The results of the analyses were robust.

The main strengths of this trial are the randomized study design, the relatively large sample size for a single-center interventional trial on OM, the implementation of musicians’ medicine, including a job-specific subjective assessment, treatment and outcome measurements, the high patient adherence rate, the long follow-up, and the comprehensive range of patient-reported outcomes, including neck pain, neck pain disability, quality of life, perceived stress, medication intake, and job-specific parameters. Furthermore, we considered health economics parameters. Additionally, validated and widely accepted clinical outcome measures were used, including the VAS for pain,^41^ NDI,^46,47^ VAS for stress^55^ and SF-12.^56–59^ In this RCT, we aimed to answer a research question with high personal relevance for adult musicians, as cNP is common in adult violinists and violists, including music students. The OM treatment was performed by an osteopath, who was also a medical doctor and orthopedic surgeon, by applying the usually performed individualized diagnosis-related osteopathic treatment.

However, the study also has limitations. This study employed a single-center setting, with the involvement of only one therapist with very specific training, and there was a high percentage of women in our study population; these factors clearly limit the generalizability of our results. The calculated sample size for the primary outcome parameter was not achieved; however, the difference in the primary outcome between the treatment groups was larger than expected. The study design had more potential sources of bias: participants in the OM group received more time and attention than those in the CG, and the blinding of patients or the therapist with regard to group allocation was not feasible within the study design. The patients themselves assessed the outcomes with patient-reported outcome measures; therefore, blinding of outcome assessors was not feasible. The primary outcome, neck pain, was measured subjectively. The lack of additional blinded objective outcome parameters is a limitation of the study. However, recently, the importance of blinding in RCTs has been discussed, as a meta-epidemiological study found no evidence for an average difference in estimated treatment effects between trials with and without blinded patients, healthcare providers, or outcome assessors.^74^ Nonetheless, the lack of blinding of outcome assessors could have led to an overestimation of the treatment effects. We used mostly validated measurement tools but also included nonvalidated tools, such as the assessment of the intake of analgesics in a diary, the assessment of days with restrictions in daily routine and the assessment of changes in complaints by patients and the rating of the effectiveness of OM treatment. These results must be considered orienting and hypothesis generating, and must be interpreted with caution. There is some discussion in the literature on how to best analyze the VAS. One analysis for example including more than 200 patients concluded that VAS might be nonlinear and thus ordinal and should be analyzed as such.^75^ However, this can be specific to the data at hand and after checking the data in our study, we saw no reason to not use ANCOVA. It is considered to be a robust method, and many researchers such as Heller *et al*.^76^ and Philip^77^ recommend parametric analyses methods as the pragmatic choice with equal power. Further, in our study the ANCOVA was also the predefined analysis strategy, which should be followed as closely as possible to minimize the possibility of bias.

Another limitation is that a number of patients in both groups did not adhere to the study protocol and used additional treatments during the first 12 weeks. However, the results were robust with respect to the sensitivity analyses regarding the PP population.

We considered the relatively short period of 12 weeks for an intergroup comparison necessary to recruit patients and ensure compliance in the CG. If the period between treatments had been longer, approximately 3–4 weeks, as is often the case in OM, we would have expected a stronger improvement in pain and function.

From a health economics point of view, the relatively short period is a limitation. If the period of the intergroup comparison had been longer than 12 weeks, it might have been possible to obtain more robust results with respect to the cost-effectiveness analyses. Another limitation is that adverse events were not assessed by patients in diaries but by interviews and reports to the study center. This could have led to an underreporting of adverse events, especially mild adverse events, because patients might have forgotten to report these events at the time of the interviews.

To the best of our knowledge, this was the first study to compare OM with no treatment option in adult violinists and violists with cNP. We found only one nonrandomized clinical trial that was a thesis for a Bachelor’s degree in the British College of Osteopathic Medicine treating 23 healthy violinists with one strain–counterstrain session, as a technique applied in OM, in comparison with positive visualization reporting improved range of motion in the OM group. This thesis showed that nine musicians felt calmer and more relaxed after the strain–counterstrain treatment, and 19 musicians felt calmer and more relaxed after positive visualization.

Regarding other therapy options in adult violinists and violists, one pre–post study with a crossover design investigated scapula taping while playing the instrument in eight professional orchestra musicians.^78^ The authors found no benefit of scapula taping regarding pain during violin playing. Other randomized trials investigating therapy options for musculoskeletal pain in a variety of adult musicians reported some improvement after exercise^79,80^ or Tui Na treatment^81,82^ but no clear benefit after yoga.^83^

To our knowledge, this was also the first study investigating the costs and cost effectiveness of OM in musicians with cNP. Our results tended to be in favor of OM but were not statistically significant. This conclusion is consistent with the literature. Steel *et al*.^32^ stated that despite some positive findings, published comparative effectiveness and health economics studies of OM are of insufficient quality and quantity to inform policy and practice. However, OM was reported to be a cost-effective strategy in patients with neck pain when compared with usual care, although it involved additional costs.^31^ In former publications,^33,34^ the cost–utility analysis identified reported improvements in pain and quality of life in patients with neck or back pain at a cost of £3760 per QALY gained.^32^

Future studies should investigate efficacy by investigating specific therapeutic effects of OM in comparison with a sham procedure and with other effective therapy methods. Possible sham procedures could include nonspecific light touch procedures in patients naïve to osteopathic treatment. The nonspecific touch procedures should include the whole body and be applied by nonosteopaths. Regarding the comparison with other effective therapy methods, these could be single therapies, such as physiotherapy and analgesics, or could include multimodal approaches. Blinding of study patients, outcome assessors and statisticians should be considered in future trials, especially if a sham procedure is developed. A future trial on OM should include multiple centers, therapists with different levels of training, a comparison with other best care options, and a more balanced sample regarding sex. Adverse events should be reported by patients in diaries.

## Conclusion

The results of this study suggest that OM might be effective in reducing the pain intensity in adult violinists and violists, including music students, with nonspecific cNP. Nevertheless, in terms of cost effectiveness, OM treatment was not superior to no OM treatment during a 12-week observation period. Further multicenter studies should investigate the efficacy of OM in comparison with an OM sham procedure and the effectiveness of OM in comparison with other therapy methods.

## Supplemental Material

sj-pdf-1-tab-10.1177_1759720X20979853 – Supplemental material for The effect of osteopathic medicine on pain in musicians with nonspecific chronic neck pain: a randomized controlled trial Click here for additional data file. Supplemental material, sj-pdf-1-tab-10.1177_1759720X20979853 for The effect of osteopathic medicine on pain in musicians with nonspecific chronic neck pain: a randomized controlled trial by Gabriele Rotter, Isabel Fernholz, Sylvia Binting, Theresa Keller, Stephanie Roll, Benjamin Kass, Thomas Reinhold, Stefan N. Willich, Alexander Schmidt and Benno Brinkhaus in Therapeutic Advances in Musculoskeletal Disease

sj-pdf-2-tab-10.1177_1759720X20979853 – Supplemental material for The effect of osteopathic medicine on pain in musicians with nonspecific chronic neck pain: a randomized controlled trial Click here for additional data file. Supplemental material, sj-pdf-2-tab-10.1177_1759720X20979853 for The effect of osteopathic medicine on pain in musicians with nonspecific chronic neck pain: a randomized controlled trial by Gabriele Rotter, Isabel Fernholz, Sylvia Binting, Theresa Keller, Stephanie Roll, Benjamin Kass, Thomas Reinhold, Stefan N. Willich, Alexander Schmidt and Benno Brinkhaus in Therapeutic Advances in Musculoskeletal Disease

sj-pdf-3-tab-10.1177_1759720X20979853 – Supplemental material for The effect of osteopathic medicine on pain in musicians with nonspecific chronic neck pain: a randomized controlled trial Click here for additional data file. Supplemental material, sj-pdf-3-tab-10.1177_1759720X20979853 for The effect of osteopathic medicine on pain in musicians with nonspecific chronic neck pain: a randomized controlled trial by Gabriele Rotter, Isabel Fernholz, Sylvia Binting, Theresa Keller, Stephanie Roll, Benjamin Kass, Thomas Reinhold, Stefan N. Willich, Alexander Schmidt and Benno Brinkhaus in Therapeutic Advances in Musculoskeletal Disease

sj-pdf-4-tab-10.1177_1759720X20979853 – Supplemental material for The effect of osteopathic medicine on pain in musicians with nonspecific chronic neck pain: a randomized controlled trial Click here for additional data file. Supplemental material, sj-pdf-4-tab-10.1177_1759720X20979853 for The effect of osteopathic medicine on pain in musicians with nonspecific chronic neck pain: a randomized controlled trial by Gabriele Rotter, Isabel Fernholz, Sylvia Binting, Theresa Keller, Stephanie Roll, Benjamin Kass, Thomas Reinhold, Stefan N. Willich, Alexander Schmidt and Benno Brinkhaus in Therapeutic Advances in Musculoskeletal Disease
