# Supplementary material for: The effect of osteopathic medicine on pain in musicians with nonspecific chronic neck pain: a randomized controlled trial
Source: Ther Adv Musculoskelet Dis. 2020 Dec 10;12:1759720X20979853. doi: 10.1177/1759720X20979853 (PMC7734566; doi:10.1177/1759720X20979853)
Supplement: sj-pdf-1-tab-10.1177_1759720X20979853 – Supplemental material for The effect of osteopathic medicine on pain in musicians with nonspecific chronic neck pain: a randomized controlled trial [file sj-pdf-1-tab-10.1177_1759720X20979853.pdf]

Please indicate your average neck pain for the last week:
Please put a cross on the scale

no pain at all **worst imaginable pain**

Supplement 1: Figure visual analog scale (VAS) pain, English translation
