# Supplementary material for: The effect of osteopathic medicine on pain in musicians with nonspecific chronic neck pain: a randomized controlled trial
Source: Ther Adv Musculoskelet Dis. 2020 Dec 10;12:1759720X20979853. doi: 10.1177/1759720X20979853 (PMC7734566; doi:10.1177/1759720X20979853)
Supplement: sj-pdf-2-tab-10.1177_1759720X20979853 – Supplemental material for The effect of osteopathic medicine on pain in musicians with nonspecific chronic neck pain: a randomized controlled trial [file sj-pdf-2-tab-10.1177_1759720X20979853.pdf]

Supplement 2: Details Handout musicians' medicine consultation - English translation

Practice

- Pay attention to healthy exercise behavior. This includes:
- Do not practice when you are tired.
- Warm yourself up before exercising, pay attention to the room temperature.
- Create an ergonomically favorable environment (e.g. regarding chair, music stand)
- Pay attention to have an upright posture and to apply an optimal playing technique.
- Change your posture between sitting and standing.
- If possible, play a varied repertoire that includes pieces that you enjoy.
- Start with small practice units according to your state of health, even if it should only be 5 minutes. Extend the practice duration over time if appropriate.
- Allow yourself a break of 10-15 minutes after 45 minutes at the latest.
- During the practice break, put down your instrument and move around.
- Practice as continuously as possible, do not suddenly increase your practice time significantly.

General recommendations

- Do endurance sports, preferably at least 3 times a week for 20 minutes (e.g. jogging, swimming, walking).
- Learn/use techniques for improved body awareness (e.g. Alexander technique, Feldenkrais).
- Learn/use a relaxation method to cope with stress (e.g. autogenic training, muscle relaxation according to Jacobsen).
- Pay attention to a generally healthy lifestyle (e.g. eat regularly, sleep regularly and sufficiently).
- Try to reduce psychological stressors and to reduce stress.
- Maintain a positive relationship with your body and your instrument.

At your workplace, pay attention as far as possible to

- Work equipment (instrument, back-friendly seating, music stands, sheet music)
- Working environment (hearing protection, light, room temperature, no draught)
- Work organization (working hours, sufficient breaks, scheduling of rehearsals, concerts, teaching, or tours)
