# Supplementary material for: The effect of osteopathic medicine on pain in musicians with nonspecific chronic neck pain: a randomized controlled trial
Source: Ther Adv Musculoskelet Dis. 2020 Dec 10;12:1759720X20979853. doi: 10.1177/1759720X20979853 (PMC7734566; doi:10.1177/1759720X20979853)
Supplement: sj-pdf-3-tab-10.1177_1759720X20979853 – Supplemental material for The effect of osteopathic medicine on pain in musicians with nonspecific chronic neck pain: a randomized controlled trial [file sj-pdf-3-tab-10.1177_1759720X20979853.pdf]

Supplement 2: Details Handout musikermedizinische Beratung

Übepraxis

- Achten Sie auf gesundes Übeverhalten. Hierzu zählen:
- Üben Sie nicht, wenn Sie ermüdet sind.
- Wärmen Sie sich vor dem Üben auf, achten Sie auf die Raumtemperatur.
- Schaffen Sie sich eine ergonomisch günstige Umgebung (z.B. hinsichtlich Stuhl, Notenständer)
- Achten Sie auf eine aufrechte Körperhaltung und eine optimale Spieltechnik.
- Wechseln Sie die Körperhaltung zwischen Sitz und Stand.
- Spielen Sie möglichst ein abwechslungsreiches Repertoire, welches Stücke mit einschließt, die Ihnen Freude bereiten.
- Beginnen Sie entsprechend Ihres Befindens mit kleinen Übeeinheiten, selbst wenn es nur 5 Minuten sein sollten. Verlängern Sie im Laufe der Zeit ggf. die Übezeiten.
- Erlauben Sie sich nach spätestens ca. 45 Minuten jeweils eine Übepause von ca. 10-15 Minuten.
- Legen Sie in der Übepause ihr Instrument ab und bewegen Sie sich.
- Üben Sie möglichst kontinuierlich, erhöhen Sie nicht plötzlich deutlich die Übezeit.

Allgemeine Empfehlungen

- Betreiben Sie Ausdauersport, am besten mindestens 3 mal wöchentlich 20 Minuten (z.B. Joggen, Schwimmen, Walking).
- Erlernen/ verwenden Sie Techniken zur verbesserten Körperwahrnehmung (z.B. Alexandertechnik, Feldenkrais).
- Erlernen/ verwenden Sie eine Entspannungsmethode zur Stressbewältigung (z.B. Autogenes Training, Muskelrelaxation nach Jacobsen).
- Achten Sie auf eine allgemein gesunde Lebensweise (z.B. essen Sie regelmäßig, schlafen Sie regelmäßig und ausreichend).
- Versuchen Sie, psychische Stressoren zu verringern und Stress abzubauen.
- Bewahren Sie eine positive Beziehung zu Ihrem Körper und Ihrem Instrument.

Achten Sie an Ihrem Arbeitsplatz soweit es Ihnen möglich ist auf

- Arbeitsmittel (Instrument, rückengerechte Sitzgelegenheit, Pulte, Notenmaterial)
- Arbeitsumgebung (Gehörschutz, Licht, Raumtemperatur, keine Zugluft)
- Arbeitsorganisation (Arbeitszeiten, ausreichende Pausen, zeitliche Einteilung von Üben, Proben, Konzerten, Unterrichten oder Tourneen)
