# Supplementary material for: The effect of osteopathic medicine on pain in musicians with nonspecific chronic neck pain: a randomized controlled trial
Source: Ther Adv Musculoskelet Dis. 2020 Dec 10;12:1759720X20979853. doi: 10.1177/1759720X20979853 (PMC7734566; doi:10.1177/1759720X20979853)
Supplement: sj-pdf-4-tab-10.1177_1759720X20979853 – Supplemental material for The effect of osteopathic medicine on pain in musicians with nonspecific chronic neck pain: a randomized controlled trial [file sj-pdf-4-tab-10.1177_1759720X20979853.pdf]

Table 4: Complaints due to musculoskeletal pain and effectiveness assessment of osteopathic medicine treatment over time

		n*	OM group n = 28 n (%)	Control group n = 34 n (%)
**Complaints due to musculoskeletal pain rated by patient, changes within the last 6 weeks**				
After 6 weeks	Completely disappeared	61	0	0
	Clearly reduced		12 (42.9)	4 (12.1)
	Slightly reduced		11 (39.3)	6 (18.2)
	Unchanged		4 (14.3)	20 (60.6)
	Worsened		1 (3.6)	3 (9.1)
After 12 weeks	Completely disappeared	61	4 (14.3)	0
	Clearly reduced		17 (60.7)	3 (9.1)
	Slightly reduced		6 (21.4)	9 (27.3)
	Unchanged		0	17 (51.5)
	Worsened		1 (3.6)	4 (12.1)
After 26 weeks (control group receiving OM intervention)***	Completely disappeared	58	2 (7.7)	0
	Clearly reduced		9 (34.6)	8 (25.0)
	Slightly reduced		7 (26.9)	13 (40.6)
	Unchanged		4 (15.4)	4 (15.4)
	Worsened		4 (15.4)	3 (9.4)
After 52 weeks (control group receiving OM intervention)***	Completely disappeared	57	1 (3.7)	1 (3.3)
	Clearly reduced		11 (40.7)	6 (20.0)
	Slightly reduced		5 (18.5)	10 (33.3)
	Unchanged		4 (15.4)	4 (15.4)
	Worsened		4 (15.4)	3 (9.4)
**Effectiveness assessment of OM treatment rated by patient, with reference to the last 6 weeks**				
After 6 weeks	Highly effective	28	9 (32.1)	**
	Effective		14 (40.0)	**
	Slightly effective		5 (17.9)	**
	Not effective		0	**
After 12 weeks	Highly effective	28	13 (53.6)	**
	Effective		11 (39.3)	**
	Slightly effective		2 (7.1)	**
	Not effective		0	**
After 26 weeks (control group receiving OM intervention)***	Highly effective	46	14 (53.9)	4 (20.0)
	Effective		8 (30.8)	12 (60.0)
	Slightly effective		4 (15.4)	4 (20.0)
	Not effective		0	0
After 52 weeks (control group receiving OM intervention)***	Highly effective	41	15 (55.6)	4 (28.6)
	Effective		6 (22.2)	5 (35.7)
	Slightly effective		5 (18.5)	5 (35.7)
	Not effective		1 (3.7)	0

* number of participants answering the question, ** does not apply, *** after 12 (12 to 26) weeks n = 28 participants of control group started to get also OM treatment (waiting list design)

n = absolute number, OM = osteopathic medicine
